# The Clinical Examination of the Chest

**Published:** 1910-04-02

**Authors:** S. H. Habershon


### HOSPITAL CLINICS.

### THE CLINICAL EXAMINATION OF THE CHEST.

By S. H. HABERSHON, M.D., F.R.C.P.

(A Lecture delivered at the Hospital for Consumption and Diseases of the Chest, Brompton, on Wednesday, February 2, 1910.)

GENTLEMEN,—In the last demonstration that I had the privilege of giving to you there was one part of the examination of the chest that I did not mention—but we cannot leave it out of notice because of its great importance—and that is the examination of the heart. In a hospital of this kind we pay special attention to pulmonary tuberculosis, and sometimes very little attention is paid to the area of cardiac dulness. Therefore it will not be out of place if I lay a little emphasis on the importance of carefully mapping out the cardiac dulness. And I think the superficial cardiac area is of more value than the deep in our investigations. Remember that it is easier to examine a heart in the areas of superficial dulness with the patient in the erect posture than in the recumbent. Especially where the lungs are emphysematous, the posterior parts do not move so freely, and I have often verified the fact that the sternal edges in the anterior parts of the upper lobes of the lung expanded more in the recumbent position, partly covering the cardiac dulness and diminishing it. As you are well aware, even in the early cases, there may be a rapid contraction of the upper lobe, both uncovering the heart and drawing it up; and this fact of contraction is of great importance in prognosis, because, if there is rapid contraction it is an evidence of Nature's effort at the healing process. It may be drawn to the third space, or even a little higher. In the case of contraction of the right lung the heart may be drawn completely across the sternum, so that the right margin of cardiac dulness extends one or two finger-breadths or further beyond the sternum. Remember also the differences in the cardiac area in children. In them the natural superficial cardiac area extends up to the third costal cartilage, instead of to the fourth, as in the adult, and the apex is very near the nipple, either in the fourth space or behind the fifth rib, whereas in the adult it is in the fifth space, within the clavicular line. With regard to the nomenclature of the apex of the heart, I think it is advisable to use the term "nipple line" only in regard to men, because in women it is such a variable quantity, especially if the breast is loose or pendulous. In women I think the term we should use is the "mid-clavicular line."

Now we come to the question of *early pulmonary tuberculosis*. In our examination of some of the cases I brought forward at the last lecture we saw some of the physical signs of early tuberculosis, but I was unable to arrange them with any method. Before dealing with the special physical signs, I want to say a word about the paths of entrance of tubercle. By the first Royal Commission on Tuberculosis it was clearly proved, after feeding experiments, that there are two common modes of entrance of tubercle. One is through the tonsils, and the other through some part of the intestinal tract. You know that in this hospital we find it very difficult indeed to trace the source of entrance, because when the case comes to autopsy it is generally one of advanced disease; therefore it is only in a very small proportion of cases—those of acute disease rapidly dying—that we are able to detect the source of entrance. In the feeding experiments on animals it was clearly shown that sometimes the tonsils were themselves affected with ulceration, and secondarily the cervical chain of glands. But sometimes tubercle was found in the cervical chain, and the tonsils themselves, though they appeared to transmit it, were not themselves invaded by tubercle. But the common event is, even though the tonsils have been the source of entrance, to find only the cervical chain affected. In our museum specimens there are many instances of tuberculous ulceration of the tonsils, some of which may be primary, but I think the greater number are secondary. It has been conclusively proved that tubercle can spread in the contrary direction to the lymph current. It may spread upwards and downwards along the walls of the lymphatic tract, so that the invasion gradually extends into the mediastinal glands, and especially into the inter-tracheal group, and from thence through the lymphatics into the lung itself. The other path of entrance is undoubtedly the intestinal tract, and in one or two acute cases of tubercle, especially in children, we have found, just as some of the Commissioners in the Royal Commission on Tuberculosis found in their feeding experiments, some small ulcers in the ileum or cæcum; more rarely in the colon, with a very distinct invasion of the sub-peritoneal lymphatics; and thence the abdominal lymphatic glands have been affected and the disease has spread, and either caused a general tuberculosis or only a pulmonary tuberculosis. There is a third path of entrance, and that is through the lungs, by inhalation, and I have no doubt some of those cases where families have been shown to have lived in a room from which the dust has not been removed, or which has not been carefully cleansed and disinfected every few years, and where one family after another dwelling there has been found to have tuberculosis, the source of entrance is probably by inhalation. And in some cases dried sputum is the source of infection. Probably when it is due to inspiration the tubercle does not directly invade the lung, but passes through the lymphatics. We may add a fourth method of invasion, but it is not a primary one. I refer to the cases of acute miliary tuberculosis. These often follow hæmoptysis, and, owing to the rupture of a vessel in the neighbourhood of a tuberculous focus, I have very little doubt that some tuberculous material is drawn on into the general circulation. Dr. Percy Kidd has shown that the walls of the minute blood-vessels may be invaded with tubercle, which may ulcerate and cause a general infection with acute miliary tuberculosis. Some years ago, at a meeting of either the Clinical or the Pathological Society, he showed some beautiful specimens illustrating the invasion of the walls of the blood-vessels.

The situation of the physical invasion of pulmonary tuberculosis has been shown to be a little below the apices of one of the lobes of the lungs. Dr. Fowler, in an extremely able paper some years ago, showed that, as a rule, tubercle commences one and a half to two inches below the summit of the upper lobes; that is to say, in the first intercostal space on one side or the other; and that this disease spreads either downwards towards the second space, or backwards into the supraspinous fossa. But Dr. Fowler also showed that sometimes the disease does not begin in the upper lobes, but in a similar situation, one and a half to two inches below the summit of the *lower lobes* on either side, and thence extends along the course of the septum. That is a thing we have to be extremely wary of, because, on examining the apices of the upper lobes, one is apt to conclude that that is always the first and earliest position of the invasion of tubercle. But we find a very distinct series of cases in which the disease commences in the lower lobe. I was going round my wards two months ago when a gentleman, who is a practitioner, accosted me and said he had been alarmed to find that he had suddenly had an attack of hæmoptysis that morning. I took him into a private room and examined his lungs, and I could find nothing wrong with the apices of the upper lobes. But there was a patch at the apex of the left lower lobe, just below the extreme summit, where expiration was distinctly prolonged, and there were a few crepitations. The signs developed somewhat, but I am glad to say are now subsiding. In this case, if one had not carefully examined the apices of the lower lobes one could easily have missed the focus of the disease.

Now as to the various modes of onset of tuberculous disease. We must remember that tubercle, uncomplicated with any mixed infection, presents a very different picture from the commoner cases that we get, where the tubercle is complicated by the invasion of other organisms. This mixed infection gives us a cause for the peculiar varieties of onset. Sometimes there is a local focus of disease which has been produced through one of the methods of invasion which I have mentioned. We find that a local focus of disease remains practically quiescent for a considerable time. In some cases the mischief goes on slowly spreading; the patient suffers not from any very active process of breaking down, nor from cough, but merely from the effect of auto-intoxication caused by the presence of this focus of disease. There is a certain group of cases that, in our old-fashioned language, we used to call cases of *decline*, and I believe that in these cases there is a distinct invasion of tubercle, in which the patient is gradually being infected by the toxins produced, but there is no rapid breakdown. There are no crepitation râles to be heard, but there may be found obscure impairment of resonance, or slight differences in the breathing. I remember in my boyhood hearing of many cases of young people who were said to be in a decline, and they differed from ordinary cases of consumption in that they did not present the stormy and acute signs of hectic fever, cough, expectoration, and very rapid emaciation; rather there was a general gradual failure of health.

The second variety is the *catarrhal*, and in the majority of cases which are presented to us there is a focus of tubercle in the lungs, and that produces a sensitive and weak spot. I believe tubercle is there from the onset; but the patient is said to suffer from incessant colds, and that is one reason why, in insurance papers, we are asked to say whether the patient is especially liable to colds on the chest. I believe these people already have tuberculous disease, and that the sensitive spot makes the patient liable to the invasion of other organisms. In this hospital you would find a large number of cases in which the history was that at a certain date they had influenza and had never been well since. That influenza was merely the stormy catarrhal onset of the disease. The entrance of tuberculosis sometimes takes place by inoculation. In this hospital, unfortunately, on several occasions, workers in our laboratory have inoculated themselves with tubercle. In one case a former resident inoculated his finger with tubercle; he was working on sputum. In another case our former laboratory man pricked his finger with a broken pen with which he used to place the sputum upon a cover slip; and both these persons in a very short time developed tubercle of the lungs. At the time the resident developed it, I remember there was an epidemic of influenza, and he was supposed to have got influenza, for he had headache and chilly feelings down his back. But one day he saw a few specks of blood in his sputum, and on examining them under the microscope he found they were almost pure cultures of bacilli. Eventually he went through sanatorium treatment, and is now completely well. Doubtless in some cases the mixed invasion is due to the *influenza bacillus*; but far more commonly it is one of the catarrhal group, such as the *micrococcus catarrhalis*. Another organism which, I think, though less commonly, is concerned in such attacks is the *diplococcus pneumoniæ*. And we have had several striking cases of that here, where the patient has gradually become well after, perhaps, several months of hectic temperature with signs of somewhat extending disease. There is also another invasion that is to be found more commonly in those cases of very acute necrotic process. Under the influence of these catarrhal organisms you will see cases, some of which I shall show you, where the tuberculous process has extended gradually, one focus after another has been formed by lymphatic spread, and you find the early signs of consolidation, and subsequently of breaking down and softening. But sometimes the softening process takes place early and rapidly, forming that group of cases which we know as *phthisis galopante*, and a more scientific term is "*acute necrosis of lung*." I have never been able to verify the mixed infection, but in some of these terrible cases we find staphylococcus and streptococcus present. One of the great advantages and uses of sanatorium treatment is to remove all these extraneous influences. In most cases we have to deal with the two great factors, the tubercle bacillus and its toxic conditions, and the resisting power of the patient. One of the most striking features of the cases that go down to the Frimley sanatorium is that in about a fortnight's time all the mixed infections disappear from the sputum. We find in London that when you take a cover-slip preparation directly after the patient has expectorated, you will observe many of these other cocci and bacilli of unknown character. On the ground of this mixed infection several lines of treatment have been adopted, as, for example, Dr. Lees' method of a continuous antiseptic inhalation, or, again, the internal administration of an antiseptic compound such as the oil of cinnamon. Another line of treatment is the administration of chloretone, an inhalant extremely useful in all catarrhs.

I think that we might perhaps include in the large group of mixed infections those cases in which there is an extremely chronic catarrhal infection, and in which it is hard to say whether the catarrh has originated from the tuberculous process, or the tubercle followed upon the catarrh. It is extremely difficult in such cases to say which is the cart and which is the horse. The patient may have a winter cough, and on examination show signs of tubercle, either plainly or, oftener, masked by emphysema. There is another group in which, also, it is hard to say which disease precedes the other—namely, the *syphilitic* group, the cases of pulmonary tuberculosis in the syphilitic subject. I believe that the syphilis precedes the tuberculosis, but when there is an invasion of tubercle in the syphilitic subject it is found as a rule that the tuberculous disease progresses in one of two ways: either an extreme amount of fibrosis is developed—there may be a patch of fibroid lung with perhaps a caseous nodule underlying it—or the influence of the syphilis is shown undoubtedly in producing gangrene of the lung. These two classes—the fibroid and the gangrenous—I have seen among our pathological cases.

I want now to mention the different physical signs for which we may look in early tuberculosis. They are difficult cases to examine, and it requires all the skill of the physician to discover the disease at an early stage. The *hæmoptysis* in the early cases constitutes a very great danger to the unwary. There are some cases—I may refer again to the insurance companies—which are covered by the phrase "it came from the throat." That is a very dangerous diagnosis to make, and insurance companies are oftener trapped by that error than by any other. I recall one man who was passed by the medical officer of the insurance company because he was thought only to be suffering from this hæmorrhage coming from the throat, and in two years' time his death claim came before the company. There were no physical signs of tuberculosis, and yet in all probability there was an early focus of disease. The very earliest sign is some slight change in the respiration of the lung. You will see, therefore, how very important it is that the person to be examined should stand in the erect position, on both feet, hands at his sides, body in perfect symmetry, and also that he should not deceive us by his breath. He should breathe as silently and softly as possible, making no panting noise. There are some difficulties in the way of examination. One of these is the stupidity or obstinacy of the patient, who will do anything except breathe naturally and quietly; and another difficulty is due to the *muscular souffle* or *susurrus*—the sound caused by the fibrillary contraction of the muscles. If we rub the finger and thumb slowly together, particularly when the skin is somewhat dry, we shall discover a creaking sound which is similar to the creaking sound one hears in the patient's breath. This masks the delicate variations which mean so much to us. The next thing to observe is an alteration of the respiratory murmur. One of the commonest of these is known as "*wavy breathing*." There are two kinds of wavy breathing. The first is due to a voluntary act, or, if involuntary, at any rate in some degree under the control of the person, such as the sobbing respiration of a child. This is one kind of wavy breathing which is seen in early pulmonary tuberculosis. The other kind of wavy breathing is rhythmic, and may be described as cardio-pulmonary respiration.

Now let us consider the character of the *expiration*. A sign of tuberculosis is the greater audibility of the expiration at the left apex than at the right. This, with high pitch (always providing the patient is not making a noise with his mouth), should give rise to profound suspicion. The breathing at the left apex normally ought to be less audible than the right.

Another feature to look for is weakened breathing. *Weak breathing* is an extremely important sign of early pulmonary tuberculosis. It is much more difficult to decide whether the weakened breathing is a sign of disease when it occurs at the left apex. Normally the expiratory breathing should be less marked on the left than on the right. A *coarse roughened* breathing, without being bronchial, is the next thing that we have to listen for. It is a physical sign of considerable significance in early pulmonary tuberculosis. An early deposit of tubercle may produce the wavy breathing, the weak breathing, or the coarse respiration; but all of these, unfortunately, may be masked by the muscular sounds to which I have already referred and which cause us so much trouble.

To discover the signs of *early consolidation* is an easier task. In these cases a certain high-pitched note characterises the expiration. We must be careful not to be deceived by an *inspiratory* high-pitch. The inspiratory high-pitch belongs to the group of cases of bronchial spasm, asthma, etc. *Bronchial breathing* is exhibited only when the expiration is high-pitched, and it is always associated with an exaggerated voice resonance. We may have anything now between broncho-vesicular breathing, when the expiration is slightly high-pitched, to well-marked bronchial or to broncho-cavernous breathing, all of them representing various degrees of consolidation. Then, of course, we shall have an increase of voice vibrations. Besides crepitant sounds, we may have any variety of crackling and creaking sounds in the pleuritic type of disease.

In considering the signs of consolidation, naturally we shall look also to deficiency of movement, which may be due either to the consolidation itself or to the accompanying pleurisy, which limits the movements of the patient's chest and makes the patient refrain from breathing. Let me impress upon you the necessity of standing your patient whenever possible with his back to the door, so as to bring out the refinements of dulness, and aid the discovery of the loss of resonance at an early stage.

